# Effects of Interventions to Improve Access to Financial Services for Micro, Small, and Medium‐Sized Enterprises in Low‐ and Middle‐Income Countries: An Evidence and Gap Map

**DOI:** 10.1002/cl2.70061

**Published:** 2025-09-10

**Authors:** Nina Ashley O. Dela Cruz, Alyssa Cyrielle B. Villanueva, Lovely Tolin, Sabrina Disse, Robert Lensink, Howard White

**Affiliations:** ^1^ Center for Evidence‐Based Social Science of Lanzhou University Lanzhou China; ^2^ Campbell Collaboration Meycauayan City Bulacan Philippines; ^3^ Campbell Collaboration Quezon City Philippines; ^4^ Campbell Collaboration Cologne Germany; ^5^ University of Groningen Groningen the Netherlands

## Abstract

Micro, small, and medium‐sized enterprises (MSMEs) account for most firms in most economies, particularly in developing nations, and are key contributors to job creation and global economic development. However, the most significant impediment to MSME development in low‐ and middle‐income countries is a lack of access to both investment and working capital financing. Due to a lack of essential track record, appropriate collateral, and credit history, MSMEs are frequently denied business loans by traditional lending institutions. In addition, MSMEs face institutional, structural, and non‐financial factors that further impede access to funding. To address this, both public and private sectors employ indirect and direct finance interventions to help MSMEs in developing and emerging economies enhance and increase their financing needs. Given the importance of MSMEs in the economy, a comprehensive overview and systematic synthesizing of the evidence of the effects of financial access interventions for MSMEs, capturing a wide variety of outcome variables, is useful. The objective of this evidence and gap map (EGM) is to describe the existing evidence on the effects of various interventions dedicated to supporting and improving MSMEs' access to credit, as well as the corresponding firm performance and/or welfare outcomes. An EGM is a systematic evidence product that displays the existing evidence relevant to a specific research question. To better understand the various interventions dedicated to supporting and improving MSMEs' access to credit, as well as their outcomes, we conducted electronic searches in databases using various search strings. This search strategy was supplemented with gray literature searches and systematic review citation tracking to ensure that the research team had identified a significant portion of relevant research works. We included studies that examined interventions aimed at enhancing MSMEs' access to finance in low‐ and middle‐income countries, targeting MSMEs including households, smallholder farmers and single person enterprise, as well as financial institutions/agencies and their staff. This EGM considered five types of interventions: (i) strategy, legislation and regulatory; (ii) financing systems and institutions; (iii) access facilitation; (iv) lending instruments or financial products; and (v) demand‐side programs for financial literacy. On the other hand, the EGM also covered outcome domains for policy environment, financial inclusion, firm performance, and welfare. Both impact evaluations and systematic reviews of relevant interventions for a previously defined target population were included in this EGM, whether they had experimental or non‐experimental designs. We considered studies that were completed or in progress. All eligible studies included a suitable comparison group for interventions. For practical reasons, studies were limited to papers written in English, with no restrictions by publication date. Before‐and‐after study designs with no suitable comparison group were excluded from the study, as well as literature reviews, key informant interviews, focus group discussions, and descriptive analyses. The result of our study is outlined in this study article, as well as an interactive map drawn as a matrix of various interventions improving MSMEs' access to finance and their corresponding firm performance and/or welfare outcomes. The preliminary map was produced in March 2022 and after adding supplementary research the updated map and analysis started in April 2022. The final interactive map is available online. The EGM includes 413 studies. One hundred and forty‐seven studies featured interventions that targeted multiple firm sizes, though most (379 studies) analyzed microenterprises, such as households and smallholder farmers. One hundred and nine studies analyzed small and medium enterprises, while seven studies analyzed community groups. Lending instruments/financial products are the most common form of intervention across all firm types, with microenterprises most often receiving the said financial intervention (278 studies). This is followed by systems and organizations (138 studies) that support better access to such financial products and services. Welfare outcomes have the most evidence out of all the outcomes of interest, followed by firm performance and financial inclusion. Welfare outcomes refer to economic, food security and nutrition, health, education, housing, well‐being, and gender outcomes. Among all firm types, welfare outcomes are primarily targeted at microenterprises. With 59 studies, we can say that small businesses have a significantly large number of enterprise performance outcomes. Of the 413 studies, 243 used non‐experimental or quasi‐experimental designs (mainly propensity score matching and instrumental variable approaches), 136 used experimental methods, and 34 were systematic reviews. 175 studies (43%) provided evidence from Sub‐Saharan Africa, 142 studies (35%) from South Asia, 86 studies (21%) from East Asia and the Pacific, 66 studies (16%) from Latin America and the Caribbean, 28 studies (7%) from Europe and Central Asia, and 21 studies (5%) from the Middle East and North Africa. Most of the evidence included covers low‐income (26%) and lower‐middle income countries (66%), and to a lesser extent upper‐middle‐income countries (26%). This map depicts the existing evidence and gaps on the effects of interventions to enhance MSMEs' access to financial services in low‐ and middle‐income countries. Interventions directed at microenterprises with welfare outcomes have a significant number of research outcomes in the literature. SME evaluations have looked at firm performance, with less focus on employment and the welfare effects on owners and employees, including poverty reduction. Microcredit/loans have been the focus of a large number of research papers (238 studies), indicating the field's growing popularity. However, emerging financial interventions such as facilitating access to digital financial services are relatively understudied. Additionally, 192 studies focus on rural or remote populations, 126 studies investigate interventions to the poor and disadvantaged, and 114 papers specifically address interventions targeted to women. Most of the research is conducted in Sub‐Saharan Africa (175 studies) and South Asia (142 studies), so further research in other regions could be conducted to allow a more holistic understanding of the effects of financial inclusion interventions. Future studies should look into strategy, law, and regulation interventions, as well as interventions targeted at SMEs, and examine policy and regulatory environment outcomes, as well as welfare outcomes. Interventions on the demand side and their impact on the policy and regulatory environment, as well as facilitating access, are relatively understudied.

**Abbreviations**
ADB: Asian Development Bank
ADBI: Asian Development Bank Institute
AFI: Alliance for Financial Inclusion
BBVA: Banco Bilbao Vizcaya Argentaria Research
CEGA: Center for Effective Global Action
EGM: evidence and gap map
GDP: gross domestic product
IADB: Inter‐American Development Bank
IBER: International Business & Economics Research Journal
IFC: International Financial Corporation
IMF: International Monetary Fund
IZA: Institute of Labor Economics
LMICs: low‐ and middle‐income countries
MSME: micro, small, medium‐sized enterprises
OECD: Organization for Economic Cooperation and Development
RCT: randomized controlled trial
SHG: self‐help groups
SMEs: small and medium enterprises
UNESCAP: United Nations Economic and Social Commission for Asia and the Pacific
UNU‐WIDER: United Nations University World Institute for Development Economics Research

## 1 Plain Language Summary

Microcredit and financial literacy dominate the evidence on micro, small, and medium‐sized enterprise (MSME) finance, while emerging areas like fintech remain underexplored.

### 1.1 What Is This Map About?

This evidence and gap map (EGM) is about various financial sector interventions that help improve access to finance for MSMEs. The map is comprised of five financial intervention categories: (i) strategy, legislation, and regulation; (ii) systems and institutions; (iii) facilitating access; (iv) lending instruments/financial products; and (v) demand‐side interventions. These interventions are described against four outcomes of interest: policy and regulatory environment, financial inclusion, enterprise performance, and welfare outcomes.

### 1.2 What Studies Are Included in This Map?

To be included in the map, the studies should be intervention studies, meaning there must be a specific program or intervention being offered or provided to different firm types. Only quantitative study designs are eligible, also accounting that there is a comparison group, or matched comparisons used in the empirical approach. The most recent search was conducted in June 2022, which added new studies to the map.

The map includes 413 studies, of which 34 are systematic reviews.

### 1.3 What Are the Findings From the Map?

The evidence is heavily dominated by two interventions, in particular microcredit and financial literacy or education programs, with 69 and 66 studies, respectively. Microfinance institutions often serve as the delivery channel for these interventions rather than being standalone interventions themselves.

Microcredit is often supplied for purposes other than enterprise development. Other financial inclusion interventions, such as health insurance, have health as their primary focus, rather than direct support for MSMEs.

Very few studies have been found on interventions providing equity, interest‐free bank accounts, and trade credit, while no evidence was found for digital literacy interventions.

The most studied outcomes of interest are economic outcomes, sales/revenue, profits, and exports as indicators of firm performance, as well as outcomes relating to access to financial services, as they all have more than 100 studies. Rare evidence exists on outcomes relating to policy and regulatory environment.

Overall, interventions for improving access to finance for MSMEs are quite numerous, though there is an imbalance in terms of specific interventions being examined. There are emerging and interesting topics, such as facilitating access interventions, which include financial technology that can be explored in the future.

### 1.4 What Do the Findings of This Map Mean?

The map is a significant material for the upcoming review on financial literacy interventions, which the team has also completed. This map also determines project directions and operational agenda to determine program priorities by identifying the evidence that exists and gaps that can be explored further. While the map does not directly assess the effectiveness of interventions, it provides a reliable source of relevant studies and systematic reviews. This can support more targeted and informed research aims going forward.

## 2 Background

### 2.1 Introduction

#### 2.1.1 The Problem, Condition, or Issue

In most economies, particularly in developing countries, MSMEs play a significant role. MSMEs make up the vast majority of businesses globally and are critical contributors to employment creation and global economic development. They account for around 90% of enterprises and two‐thirds all jobs in the globe, with formal and informal MSMEs accounting for up to 50% of national income (gross domestic product [GDP]). To accommodate the rising global workforce, it is anticipated that 600 million jobs will be required by 2030, making MSME development a top priority for many governments around the world. Thus, MSME financing is important since MSMEs are at the heart of job creation (World Bank n.d.)

However, the biggest hurdle to MSME development in low‐ and middle‐income countries (LMICs) is often a lack of access to finance for both investment and working capital (IFC 2017). The needs of MSMEs, especially micro‐enterprises, are small loans available on short notice, which the formal sector is ill‐suited to provide. Instead, many owners of MSMEs turn to internal finances or cash from friends and family to start and run their businesses. Most MSMEs are also not considered eligible for business loans by traditional lending institutions, owing to a lack of necessary track record, acceptable collateral, and credit history. Furthermore, MSMEs may be discouraged by the tedious documentation process required for business loans (Organization for Economic Cooperation and Development [OECD] 2015).

According to estimates of the SME Finance Forum (2020), 131 million firms in developing nations, or 41% of formal MSMEs, have an annual financing need of $4.5 trillion (SME Finance Forum 2020). The East Asia and the Pacific region has the highest share of the global finance gap (46%), followed by Latin America and the Caribbean (23%) and Europe and Central Asia (15%). More than half of all small and medium enterprises (SMEs), both formal and informal, lack access to formal credit (World Bank n.d.) Financing constraints are exacerbated for informal businesses, which are often small and contribute significantly to economic activity and employment despite being less productive than formal businesses. Unregistered businesses rely mostly on informal financing that, while beneficial in terms of simplifying access to capital, is linked to lower enterprise development and higher illegality.

Even though the informal sector accounts for a large portion of unmet loan demand, many businesses remain informal due to a lack of incentives and capacity to formalize. It may take a long time to create an enabling environment for firms to formalize, as it not only requires the establishment of solid institutions, laws and regulations, infrastructure, and education, but it also necessarily requires the identification of business‐oriented incentives for firms, such as access to new market opportunities and access to financial and non‐financial services, which would make it a profitable decision for firms to register their business (IFC 2013).

More generally, institutional, structural, and non‐financial factors all have a role in SMEs' inability to obtain financing. Some of these factors are (i) lack of credit information when assessing borrowers' creditworthiness; (ii) absence of alternatives to traditional collateral‐based lending; (iii) ineffective debt recovery and poor business exit mechanisms; (iv) inaccessible financing instrument alternatives; and (v) lack of technological innovation in credit assessment practices (IFC 2017). These internal and external challenges limit MSMEs' development and potential to create jobs, contribute to GDP, and aid nations' export competitiveness. The public sector has attempted to play several roles in bridging the financial access gap for SMEs. Financial inclusion programs, which aim to improve poor people's access to financial services, have been made available in low‐ and middle‐income countries to improve the welfare of poor and low‐income households. However, financial inclusion interventions appear to have unpredictable and possibly small effects (Duvendack and Mader 2019). Therefore, both the public and private sectors use indirect and direct finance interventions to improve and expand the financing needs of MSMEs in developing and emerging economies.

### 2.2 Description of the Interventions

In the literature, the policy interventions that have been implemented to alleviate the access constraint include: (i) legal and regulatory interventions; (ii) financial infrastructure components designed to address information asymmetry and reduce transaction costs; (iii) public support mechanisms; and (iv) private sector models suited to provide sustainable financial services to SMEs (IFC 2010). The public support schemes and private sector initiatives are made up of a diverse set of programs that fall into numerous categories and are further divided into sub‐categories. Examples of these various interventions to expand SME finance that have been established to directly target SMEs are establishing or expanding financial infrastructure, partial guarantee schemes, commercial banking models, and other private sector initiatives (IFC 2013). Furthermore, countries usually employ several interventions that appear to be part of a complete strategy aimed at creating a more conducive environment for SMEs by coordinating and addressing SME development initiatives in a holistic manner.

Therefore, taking these into consideration, the interventions in this EGM are divided into five categories, including: (i) strategy, legislation, and regulation; (ii) systems and institutions; (iii) facilitating access; (iv) lending instruments/financial products and services; and (v) demand‐side intervention. These MSME finance interventions have an impact on the amount of financing and/or investment available; MSME firm performance (e.g., profit, sales, assets, business expansion, indebtedness, survivability, and technology adoption); employment and wages; poverty; financial knowledge or literacy; and women entrepreneurship (Asian Development Bank [ADB] 2021).

### 2.3 How the Interventions Might Work

**Figure 1:**
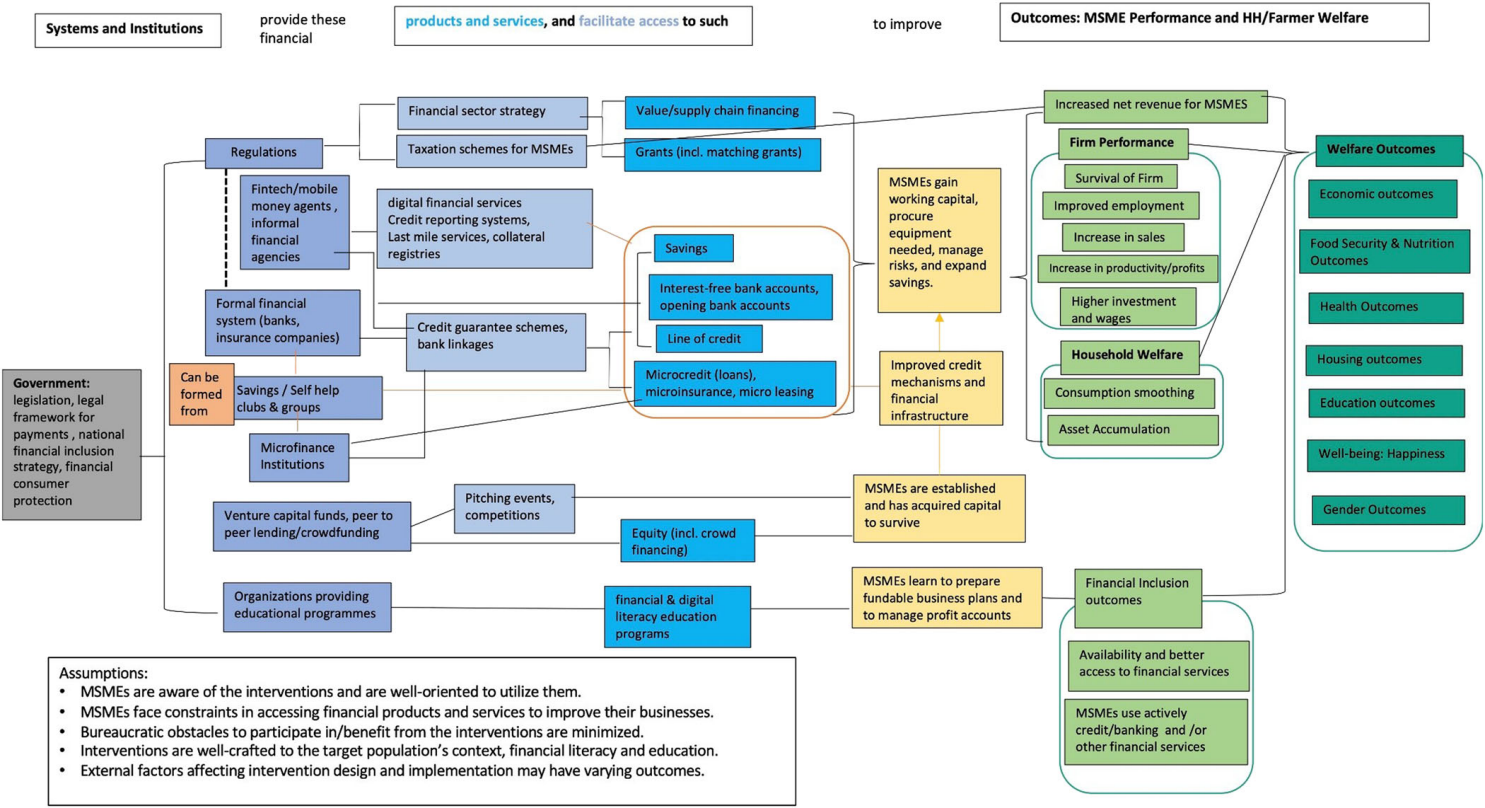
Theory of change for interventions improving access to finance.

The Theory of Change (ToC) presents how different levels of various financial interventions may improve access to finance. These interventions are shown as the three columns in different shades of blue boxes in Figure 1.

- From the government regulations providing financial sector strategy, value, or supply chain financing and grants, including matching grants, help MSMEs gain working capital, procure needed equipment, and manage risks.
- From various legal and regulatory framework surrounding formal financial institutions like banks and insurance companies, and microfinance institutions and savings or self‐help groups (SHGs), and even financial technologies, facilitate access to support MSMEs through different financial products such as savings, interest‐free bank accounts or new bank accounts, lines of credit, and microcredit (loans) as well as microinsurance and microleasing. Credit lines, microcredit (loans), and microinsurance are also typically channeled through credit guarantee schemes and bank linkages.
- Some systems, like venture capital funds, peer to peer lending, or crowd funding, organize pitching events and competitions that offer equity, including crowd financing to aid establishment or development of MSMEs.
- Some organizations providing educational programs provide financial literacy training and other educational programs to assist MSMEs in preparing fundable business plans and manage profit accounts.

As MSMEs are equipped with resources, knowledge, and platforms to improve access to finance, these in effect lead to various outcomes on policy and regulatory environment, financial inclusion, enterprise performance, and welfare.

- *Policy and regulatory environment* outcomes include inclusive finance strategies and policy and practice, regulatory framework, and institutional capacity. These outcomes refer to indicators of clear and standardized rules, policies on how financial institutions should operate, reforms to support an enabling environment, and mechanisms to develop business models for improving access to finance.
- *Financial inclusion* outcomes are comprised of financial and digital literacy, availability of financial services, access to financial services, active use of credit or banking services, and use of other financial services. These indicate that MSMEs have better access to financial services and products and are well‐oriented on how these products and services support them. These outcomes, in turn, lead to different welfare outcomes.
- *Enterprise performance* outcomes include firm survival, improved employment and wages, increase in sales and profits, increase in productivity, and higher investments. Some indicators also refer to improvement in management practices, such as expenditure on research and development and establishment or creation of firms. These firm performance measures also lead to some welfare outcomes.
- *Welfare outcomes* cover economic, food security and nutrition, health, education, housing, well‐being, and gender outcomes. Economic outcomes refer to indicators of economic activities such as investments, consumption/expenditure, growth, and poverty reduction measures. Some financial interventions to MSMEs address nutrition needs among households and firms. Health outcomes refer to indicators of health treatment, practices, and medical expenditures, including mental health measures. Housing outcomes refer to physical improvements in terms of housing. Education, as one of the measures of human development, includes measures on schooling, level of education of firm owners or even their children, attendance, and education expenditures. Well‐being outcomes can be measured using the financial worries index, happiness scale, and financial security scale. Lastly, gender outcomes refer to measures of women empowerment, women entrepreneurship, and women as decision‐makers in terms of financial management aspects.

To establish a comprehensive framework for assessing financial inclusion interventions, we developed an intervention taxonomy through an in‐depth review of the current literature and frameworks. Combining existing taxonomies, expert consultations, and thematic analysis of the literature, we have identified five intervention types. These interventions, which ranged from demand‐side initiatives to policy and regulatory measures, were meticulously intended to reflect the wide range of interventions used to improve financial access for MSMEs.

Table 1 in the methods section provides a detailed classification of the main categories and their sub‐categories. It is closely linked to the ToC, which outlines the expected pathways by which each type of intervention is expected to yield the desired outcomes. Financial literacy interventions, for example, are believed to improve the capacity of individuals to make financial decisions, which can result in improved financial inclusion and, eventually, economic well‐being.

**Table 1:** Intervention categories and sub‐categories.

Interventions	Sub‐categories	Definitions/descriptions
Strategy, legislation and regulation	National financial inclusion strategy	A set of strategies to provide individuals and businesses with access to and informed use of a wide range of high‐quality and affordable savings, credit, payment, insurance, and investment products and services that meet their needs
	Financial sector legislation and regulations	The legal and regulatory framework in a specific country that creates the rules by which all financial institutions, instruments, and markets operate
	Financial consumer protection	Rules governing liability and recourse, disclosure, and data privacy and security
Systems and institutions	Formal financial system (banks and insurance companies)	Interventions serving MSMEs to increase competition and market diversification
	Microfinance institutions	Interventions from microfinance institutions serving MSMEs (e.g., interventions that aim to ensure finances for low‐income borrowers, or empower women by financing MSMEs)
	Mobile money agents (including remittances)	Interventions supporting critical financial operations such as money transfers and bill payments by converting e‐money to cash
	Venture capital funds	Investments made during the launch stages of a business in exchange for a share in the company, usually consisting of at least 10 to 20 partners
	Peer to peer lending/crowdfunding	Lending money to individuals or businesses via online services that connect lenders and borrowers, attempting to operate at a lower cost and deliver services at a lower cost than traditional financial institutions
	Savings clubs and self‐help groups (SHGs)	Savings clubs offer reliable savings mechanisms, improvement of credit access, promotion income‐generating activities, among other activities
		SHGs pertain to groups that participate in collective bargaining, risk spreading, and peer education and social assistance, among other activities.
Facilitating access	Bank linkages	When an MSME ties with MFIs and lending institutions that could assist them in expanding their understanding of accessible financial services
	Pitching events, competitions	Contests where entrepreneurs pitch their business plans to win funding for their start‐ups, which serve as a way to help entrepreneurs evaluate their businesses, the commercialization concept, and expected revenue streams, and may include mentoring and pitch training for preparation
	Credit guarantees, including Partial Credit Guarantee Schemes	Arrangements in which a third party, known as the guarantor, agrees to repay the lender part or all of the loan amount if the borrower defaults
	Fintech/Digital financial services (mobile and e‐money and banking and payment systems and infrastructure)	Interventions that permit mobile banking, the settlement of (international) payments, and the collection and use of alternate data sources for creditworthiness assessments of SMEs, among other things
	Informal financial agencies or agents including last mile interventions	Funds sourced outside the regulatory framework and enforcement of a central banking and finance authority, such as personal savings, borrowing from relatives, or “loan sharks”
	Collateral registries	A registry where individuals and businesses can register their movable assets (such as inventory, accounts receivables, crops, and equipment) as collateral for loans to obtain credit from lenders
	Credit reporting systems	Historical credit data and other important information on large firms and individuals widely available to address information asymmetries
Lending instruments/finance products	Microcredit (loans)	Also known as microloans, or small working capital loans provided by microfinance institutions to low‐income individuals or organizations who are traditionally excluded from regular banking
	Line of credit	A contract between a bank and a borrower that establishes a maximum loan balance that the bank will allow the borrower to keep
	Savings	Deposit and savings products used as basic financial management tools to help MSMEs organize their earnings and savings
	Equity (including crowd financing)	Securities in a private enterprise (company) that is not listed on stock exchanges are sold to raise funds from the crowd (via an internet platform), similar to debt crowdfunding, but instead of raising loan cash, the company raises equity capital by selling stocks
	Grants (including matching grants)	Costs of training, marketing, and/or visiting trade fairs covered by the government
	Supply chain financing	The use of financing and risk mitigation products/tools such as contracts, receivables discounting, factoring, payables finance (reverse factoring), distributor finance, and purchase‐order finance to optimize the management of working capital and liquidity invested in supply chain processes and transactions
	Interest free bank accounts	Bank account with a zero percent interest rate
	Trade credit	Arrangements where buyers can purchase products or services on credit and pay the supplier later (i.e. a supplier provides a short‐term loan to a buyer)
	Microinsurance	Insurance provided by MFIs, cooperatives, and NGOs to small and micro entrepreneurs as a risk management tool and social protection product
	Microleasing/hire purchase	External asset‐based financing where SMEs lease an asset to generate cash flows or substitute to collateral
Demand‐side interventions	Financial literacy and education programs	Basic accounting and management information and tools supplied to MSMEs, allowing them to grow and acquire the necessary business skills and procedures for prudent and effective management
	Digital literacy	Training on how to conduct and exploit business prospects using e‐business technology to achieve higher entrepreneurial success
	Opening bank accounts for entrepreneurs	Eliminating discriminatory banking laws and simplifying account opening process/requirements

*Note:* Category on the financial sector legislation and regulation may also capture tax regime or legal and regulatory framework for payments (inc. insolvency mechanisms).

This linkage between the intervention taxonomy and the ToC ensures a holistic understanding of how each intervention type works to achieve its intended impact on policy, financial inclusion, enterprise performance, and welfare outcomes.

Furthermore, the taxonomy underscores the interconnectedness of financial interventions, with overlaps observed between various categories. Financial literacy programs and fintech solutions, for example, support both demand‐side interventions and the facilitating access categories.

Thus, we can gain a better understanding of how financial interventions contribute to desired outcomes by acknowledging the interconnectedness of these interventions and their expected pathways to impact.

### 2.4 Why It Is Important to Develop This EGM

Although MSMEs contribute substantially to employment creation and inclusive economic growth, they face structural hurdles to accessing finance, with the lack of finance for investment and working capital often cited as a preventing factor for MSME development (see e.g., IFC 2017; World Bank 2014).

There are a variety of interventions targeted explicitly at MSMEs and the obstacles they face in the traditional finance market. These interventions include supporting financial intermediaries, directly providing financial products and services, or otherwise facilitating access to them, and demand‐side interventions such as training in financial literacy.

While impact evaluation studies and systematic reviews focusing on MSME financing exist, they are often limited in scope. For instance, some systematic reviews only assess the effects of certain interventions, such as microcredit (Vaessen et al. 2014) and SHGs (Brody et al. 2015; Gugerty et al. 2019). An enlarged scope of financial interventions may be present in other studies, but the included population is restricted to either microenterprises (Cui et al. 2013) or SMEs (Kersten et al. 2017) alone. In other examples, both MSMEs may be fully covered, but the assessment is hinged on a sole outcome, such as employment creation (Grimm and Paffhausen 2015). To date, there are also no EGMs on access to finance interventions targeting MSMEs.

To address the gaps, this EGM provides a comprehensive overview and systematic synthesis of the evidence on the effects of financial access interventions for MSMEs, capturing a wide range of outcome variables. It encompasses literature on interventions aimed at increasing MSME access to finance and is followed by a review of areas of interest. Consequently, this EGM can serve as a tool for holistically assessing interventions and their outcomes while identifying further gaps in the evidence base on MSME financing. This will offer policymakers in both the public and private sectors a complete knowledge base, crucial for developing and supporting SME finance programs, which account for a significant portion of support for inclusive finance.

### 2.5 Objectives

The goal of this EGM is to describe the existing evidence on the impacts of various interventions to support and improve MSMEs' access to finance. The interventions are divided into five categories, including strategy, legislation, and regulation, systems and institutions, facilitating access, and lending instruments/financial products and services, and demand‐side interventions, as illustrated in the ToC (Figure 1). Consequently, such interventions lead to fundamental outcomes, which include firm‐level outcomes and welfare outcomes, such as economic, food security and nutrition, health, housing, education, well‐being, and gender.

The map's specific objectives are as follows:

i. Establish a clear definition of the interventions and outcomes related to interventions to improve access to finance interventions of MSMEs;
ii. Map the studies on the impact of the interventions on MSMEs' access to financing projects/programs based on primary studies and systematic reviews of such studies; and
iii. Provide a descriptive overview of interventions, contexts, study designs, and geographical distribution of studies.

This project generates an online and interactive EGM across various interventions improving MSMEs' access to finance and their corresponding firm performance and/or welfare outcomes. The preliminary map was produced in March 2022 and, after adding supplementary research, the updated map and analysis started in April 2022.

### 2.6 Snapshot of the MSME Finance EGM

**Figure 2:**
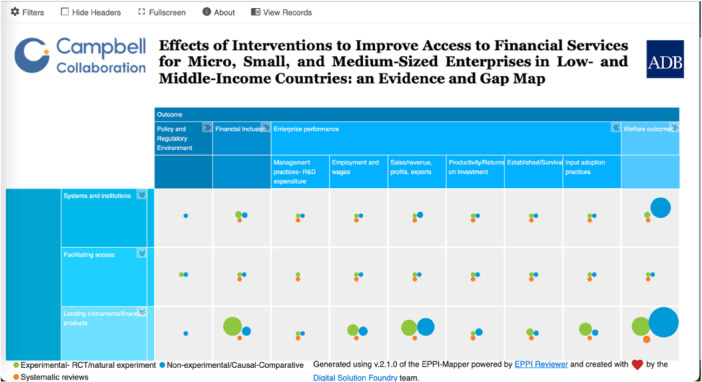
Snapshot of the MSME finance map.

The EGM showcases the matrix of financial interventions against outcomes, with study design as the segmenting attribute shown as bubbles (Figure 2). Green represents experimental study design; blue represents non‐experimental study design; and orange represents systematic reviews. The size of the circle is determined by the volume of studies in that cell. This interactive map also presents various filters as secondary dimensions: study design, country by income, country by region, firm sectors, population sub‐groups, scope, and firm types.

## 3 Methods

### 3.1 EGM: Definition and Purpose

#### 3.1.1 Defining EGMs

An EGM is a systematic evidence product that displays the available evidence relevant to a specific research question. It is a systematic presentation of the availability of relevant evidence for a particular policy domain (Saran and White 2018). Like systematic reviews, an EGM subscribes to a pre‐specified and published protocol, but it only provides a summary of the evidence. It does not assess the findings from the studies covered. Hence, an EGM covers a wider scope than systematic reviews.

The final output of an EGM is a research article or report, but conveniently, it can also be disseminated through an interactive map plotted as a matrix of included studies and their corresponding interventions and outcomes. At first sight, one can check the evidence available or lack thereof for one's particular subject matter. This EGM on improving access to finance for MSMEs includes evidence from impact evaluations and systematic reviews. Studies may cover multiple firms, interventions, and outcomes, and hence may appear in multiple cells.

#### 3.1.2 Type of Population

The map includes interventions in low‐ and middle‐income countries targeting the following population^1^: (i) MSMEs, including studies focusing on households, smallholder farmers, and single‐person enterprise or business owners; and (ii) financial institutions and agencies, and the staff of those institutions and agencies. The population also includes individuals who are unable to start and operate their businesses due to both financial and non‐financial constraints.

#### 3.1.3 Types of Interventions/Problem

The EGM considers five types of interventions that aim to: (i) deliver strategy, legislation and regulatory aspects; (ii) enable financing through systems and institutions; (iii) facilitate access to finance; (iv) deliver different lending instruments or financial products, including traditional forms of microcredit; and (v) provide demand‐side interventions such as programs on financial literacy. Table 1 shows the resulting set of intervention categories.

#### 3.1.4 Types of Outcome Measures

Table 2 presents the main outcome categories and sub‐categories. Outcomes included in the EGM are based on the ToC that is presented in this report.

**Table 2:** EGM outcomes.

Outcomes	Sub‐categories
Policy and regulatory environment	Inclusive finance strategies and policy and practice
	Regulatory framework
	Institutional capacity
Financial inclusion	Financial and digital literacy
	Availability of financial services
	Access to financial services
	Active use of credit/banking services (including loan volume)
	Use of other financial services
Enterprise performance	Management practices, R&D expenditure
	Employment and wages
	Sales/revenue, profits, exports
	Productivity/Returns on Investment
	Established/Survival
	Input adoption practices
Welfare outcomes	Economic (including employment)
	Food security and nutrition
	Health
	Housing
	Education
	Well‐being: Happiness
	Gender

#### 3.1.5 Criteria for Including and Excluding Studies

#### 3.1.6 Types of Study Designs

The EGM includes impact evaluations or systematic reviews covering relevant interventions for a selected target group that fall within the above‐discussed categories. Moreover, eligible studies need to include a methodological design that addresses the potential endogeneity problem due to selection biases concerning the intervention.

Included studies either have quasi‐experimental designs or other suitable methodological approaches with a potential selection bias awareness. Methodological designs and analytical frameworks that meet this criterion are natural experiments, randomized controlled trials (RCTs), regression discontinuity analyses, propensity score matching, difference‐in‐difference approaches, and the use of instrumental variables.

Before‐and‐after study designs with no suitable comparison group and designs that include a selection of a control group without accounting for selection biases are excluded from the EGM. Qualitative evidence or study designs, including literature reviews, key informant interviews, focus group discussions, and descriptive analyses were also not considered for the map.

#### 3.1.7 Types of Evidence

This EGM included ongoing and completed impact evaluations and systematic reviews of financial inclusion for MSMEs. Studies of financial inclusion in which there is no intervention were not included. The following are the impact evaluation designs covered:

- Experimental designs: RCTs and natural experiments Non‐experimental designs: (i) quasi‐experimental designs such as propensity score matching and regression discontinuity, (ii) regression‐based designs such as instrumental variables and Heckman sample selection models; and (iii) other studies with a comparison group.

We did not include studies on ex‐ante impact estimates (e.g., cost–benefit analysis) or other types of studies without empirical application.

### 3.2 Types of Settings

Impact evaluations conducted in LMICs based on the World Bank classification are included in this EGM. Systematic reviews that do not include at least one of the LMICs are not eligible.

### 3.3 Status of Studies

We have compiled studies that are completed or still ongoing, though almost all the studies are completed. Studies are not restricted by the publication date, but are limited to papers in the English language for feasibility reasons.

#### 3.3.1 Search Methods and Sources

Table 3 outlines the databases, organizations, and journals that the team conducted searches in to collect studies for the EGM.

**Table 3:** Search sources.

Database searches	Organization searches	Journal searches
Agris Repec Academic Search ScienceDirect EconPapers Econstor Taylor & Francis Online Jstor Scielo ResearchGate Semantic Scholar Ebsco Discovery SpringerLink Emerald Insight	3ie impact evaluation repository Economic Research Institute for ASEAN and East Asia World Bank e‐library Banco Bilbao Vizcaya Argentaria (BBVA) Research International Financial Corporation (IFC) (last search February 24, 2022) International Monetary Fund (IMF) Social Protection Organization Massachusetts Institute of Technology's digital repository Innovation Growth Lab Global Partnership for Financial Inclusion Institute of Labor Economics (IZA) Center for Economic Policy Research Innovations for Poverty Action Alliance for Financial Inclusion (AFI) SME Finance Forum (last search February 24, 2022) Organization for Economic Cooperation and Development (OECD) library (last search February 23, 2022) Asian Development Bank (ADB) Asian Development Bank Institute (ADBI) Inter‐American Development Bank (IADB) Poverty Action Lab Center for Effective Global Action (CEGA) United Nations University World Institute for Development Economics Research (UNU‐WIDER) CiteSeerX National Bureau of Economic Research VoxDev	*International Business & Economics Research Journal (IBER)* *Journal of Economics and Behavioral Studies* *International Small Business* *Journal of Small Business Economics* *The Journal of Finance* *Journal of Banking and Finance* *Journal for SME Development* *Review of Development Finance* *American Economic Review* *Journal of Entrepreneurship and Small Business* *Journal of Asian Finance* *Economics and Business* *SSRN Electronic Journal* *World Development* (last search June 7, 2022) *Journal of Development Economics* *Journal of Development Studies* *Journal of Development Effectiveness* (last search June 7, 2022) *Journal of Innovation and Entrepreneurship* *American Economic Review* *Journal of Business Venturing*

An information specialist conducted the last database search on December 16, 2021, using a designed search strategy (see Appendix S1A.2).

These organization or website searches were executed for supplemental research of studies or gray literature screening from March 2022 to June 2022 (see Appendix S1A.1 for the URLs). The search terms used included combinations of the following keywords and phrases: “MSME financial access interventions,” “micro, small, and medium enterprises finance,” “financial inclusion for MSMEs,” “impact of financial services on MSMEs,” “MSME credit access,” “MSME financing solutions,” “financial services for small businesses,” “microenterprise financial support,” and “economic impact of MSME financial interventions.”

The journals were selected based on recommendations from some content experts in our team (R.L. and H.W.) and were chosen for their relevance to the interventions considered in this map. Keyword searches for each journal included the same and combinations of search terms as with the organizations and website searches. The searches were conducted using various academic databases and journal platforms where these journals are hosted. This included direct searches on journal websites, as well as through academic databases such as JSTOR, ScienceDirect, SpringerLink, and Wiley Online Library. The team executed these journal searches between April 2022 and June 2022.

In addition, we also conducted bibliographic searches, which entailed screening the systematic reviews to locate additional primary studies, such as those by Duvendack and Mader (2019). To identify additional primary studies including reviews, we have also conducted bibliographic back‐referencing of reference lists of all included systematic reviews. The team conducted these snowballing exercises from May 15 to 25, 2022.

After conducting the database searches, the results were imported into a bibliographic reference manager (Zotero). Duplicates were also managed in the EPPI‐Reviewer 4 software using its “manage duplicates” function. Here, duplicate studies sourced from multiple databases were identified and removed. Four reviewers conducted the title and abstract screening (N.A. O.D.C., A.C.B.V., L.T., and S.D.), forming two pairs, with a third‐party arbitrator for disagreements (H.W.) and N.A.O.D.C. as the arbitrator for the pair she was not part of. Full‐text screening and coding or data extraction were done by the same four reviewers, with two people from each pair to reconcile the opposite group's disagreements.

#### 3.3.2 EGM Protocol

The EGM protocol has been published on July 5, 2023, at Wiley Online Library.^2^

#### 3.3.3 Stakeholder Engagement

This EGM was produced under close collaboration with the Independent Evaluation Department (IED) of the ADB.^3^ Ari Perdana leads the IED team, which consists of Ma. Patricia Lim and Valerie Anne S. Melo‐Cabuang, under the direction of Joanne Asquith. Substantial input was provided by other IED staff: Maya Vijayaraghavan, Paolo Obias, Ambra Avenia, Alexander Welsteed, and Mitzirose Legal.

The existing EGM has undergone review and feedback by an Advisory Group comprised of the following:

1. Representatives from the Alliance for Financial Inclusion.
2. Dr. Maren Duvendack (University of East Anglia).
3. Mike Joyce (SME financial inclusion practitioner).
4. Dr. Masato Abe (United Nations Economic and Social Commission for Asia and the Pacific [UNESCAP]).
5. Yaroslava Babych (Tbilisi State University).
6. Gubad Ibadoghlu (Economic Research Center, Azerbaijan).
7. Kelly Hattel (ADB).
8. Shiu Raj Singh (ADB).
9. Daniel Suryadarma (ADB Institute).
10. Long Q. Trinh (ADB Institute).

We have also gathered colleagues from financial sector organizations and other experts/authors on the topic of MSME finance.

#### 3.3.4 Analysis and Presentation

##### 3.3.4.1 Report Structure

The report structure is an outline that includes major sections as well as tables and figures in the main body. Additional tables and figures are available as online supplements in the annexes.

##### 3.3.4.2 Dependency

We linked all publications of the same study in the EGM to count as one study (including protocols if available and any secondary analyses), with the most recent open‐source version included. When various publications from the same study report different outcomes, they are all included. It is worth noting that systematic reviews are likely to include the RCTs shown on the map, and that the same RCT may appear in multiple systematic reviews. Regardless of whether they had previously been included in a systematic review, all relevant RCTs were included.

##### 3.3.4.3 Unit of Analysis

Individual studies are the report's unit of analysis, with each item representing a mix of interventions and outcomes. The results are descriptive, displaying the distribution of research by study design, country income, country region, firm sectors, population, publication status, scope or geographical coverage, firm type, interventions, and outcomes. The aggregate map is presented in a colored bubble map showing well‐evidenced areas and low‐evidenced areas based on the size of the circles.

In cases where studies are published in multiple formats, such as working papers and journal articles, the most recent version is included in the map. For systematic reviews published in multiple versions, the Campbell review version is preferred.

The map displays intervention categories against outcome categories. Alternative versions of the map include types of firms receiving interventions against intervention categories and outcome categories, population filters against intervention and outcome categories, and country by region against intervention categories. The report describes the evidence according to these intervention categories, outcome categories, and firm types. The list of filters is described in the next subsection.

##### 3.3.4.4 Filters for Presentation

A framework for assessing the effectiveness of financial inclusion interventions has been presented by the map in the first section. The ToC provides an overview of target populations, platforms, products, systems, and outcomes, allowing for an in‐depth analysis of the intervention landscape. By aligning strategies with theoretical impact pathways makes it feasible to assess each intervention's contribution to desired outcomes systematically.

The evidence base that met our inclusion criteria is displayed as a matrix of interventions (rows) and outcomes (columns) in our EGM. Each study was assigned to the cell in which it provides evidence. Because most studies include numerous outcomes and interventions, they appear repeatedly on the map. Both the interventions and the results were divided into categories and sub‐categories. The color‐coding of bubbles at the bottom of the map indicates study designs. Each study has been linked to a weblink that takes the user to the website where the study's complete text or paper may be found. The map is interactive, so clicking a cell will pull up a list of studies in that cell, and clicking a row or column heading will pull up a list of studies in that row or column. Users of the interactive EGM can also use the following filters to narrow down studies:

1. Study design: experimental, non‐experimental, or systematic review.
2. Country by income: low‐income, low‐middle income, upper‐middle income.
3. Region: East Asia & Pacific, Latin America & Caribbean, Middle East & North Africa, North America, South Asia, Sub‐Saharan Africa, Europe & Central Asia.
4. Firm sector: agriculture; construction; manufacturing; services; transportation and communication; wholesale and retail trade; others; not reported.
5. Population: women; youth; older persons; poor and disadvantaged; humanitarian settings; people with disabilities; rural/remote; urban; both rural and urban; financial institutions and agencies and/or their staff; unclear.
6. Publication status: completed, ongoing.
7. Scope/geographical coverage: local, national, regional, unclear.
8. Types of firms: micro enterprise, community group, small enterprise, medium enterprise.

##### 3.3.4.5 Scope

The scope of the map comprises the following: (1) systems showcasing financial framework and financial institution and/or other sources of financial interventions; (2) platforms facilitating access to finance; (3) actual financial products and services; (4) the population of interest; and (5) the outcomes of interest.

###### 3.3.4.5.1 Systems and Institutions or Sources of Financial Interventions (1)

The map includes interventions from different systems and institutions, including various public and private organizations that provide financial services or products, such as governments, international donors or organizations, microfinance institutions, banks and insurance companies, and organizations that offer financial education and management programs.

###### 3.3.4.5.2 Platforms Facilitating Access (2) to Lending Instruments or Financial Products and Other Financial Services (3)

These interventions are utilized by MSMEs with the assumptions that they are well‐oriented to navigate such platforms and products designed for them and that they are facing constraints in terms of access, knowledge, and awareness.

###### 3.3.4.5.3 Population/MSMEs as Beneficiaries (4)

The target population groups included in this map are LMICs following the World Bank classification, MSMEs, households and/or single‐person enterprise owner, and farming households and/or farmers. These also include individuals or households who intended to start a business.

###### 3.3.4.5.4 Outcomes of Interest (5)

The study's outcomes of interest fall into four main categories: (i) Policy Environment, which examines at regulatory frameworks and policies pertaining to financial inclusion; (ii) Financial Inclusion, which assesses access to and usage of financial services; (iii) Firm Performance, which evaluates impacts on the growth and sustainability of MSMEs; and (iv) Welfare, which measures socioeconomic changes.

#### 3.3.5 Data Collection and Analysis

##### 3.3.5.1 Screening and Study Selection

At the title/abstract, full‐text screening stages, and coding or data extraction, we used the EPPI reviewer to assess studies for map inclusion. Title and abstract screening is performed by four reviewers (N.A.O.D.C., A.C.B.V., L.T., and S.D.), forming two pairs, with a third‐party arbitrator for disagreements (H.W.) and N.A.O.D.C. as the arbitrator for the pair she was not part of. Full‐text screening and coding or data extraction are done by the same four reviewers, with two people from each pair to reconcile the opposite group's disagreements.

##### 3.3.5.2 Data Extraction and Management

For included studies, a data extraction tool was used to capture descriptive data from impact evaluations and systematic reviews. The descriptive data includes all the necessary information required to generate the map, such as intervention type, outcome, study design, firm types, and firm sectors, among others.

The data extraction tool and coding framework are presented in Appendices S1A and S1B. A total of four reviewers conducted the data extraction independently, two reviewers per study, and two from each acted as the third‐party arbiter to resolve the disagreements. The four reviewers are trained with the tool through piloting sessions before the commencement of the coding stage. This ensures consistency in coding and the resolution of any issues or ambiguities.

##### 3.3.5.3 Tools for Assessing the Study Quality of Included Reviews

Critical appraisal was not conducted for systematic reviews and impact evaluations. However, a thematic synthesis was written for selected and included systematic reviews in the map, and study designs of the impact evaluations were collected through the data extraction tool. A narrative synthesis was originally planned in the protocol.

## 4 Results

### 4.1 Description of Studies

#### 4.1.1 Results of the Search

The records identified from the databases and registers numbered at 15,488, of which 3,211 were duplicates, resulting in a total of 12,277 studies for title and abstract screening. After title and abstract screening, 11,310 were excluded, leaving 967 studies for full‐text screening. A total of 454 records were included for coding because 513 were excluded at the full‐text screening stage, mostly due to study design and intervention studies. After coding and data extraction, 317 studies were included in the initial phase or preliminary map, while 137 were excluded due to some duplicates and wrong study designs.

**Figure 3:**
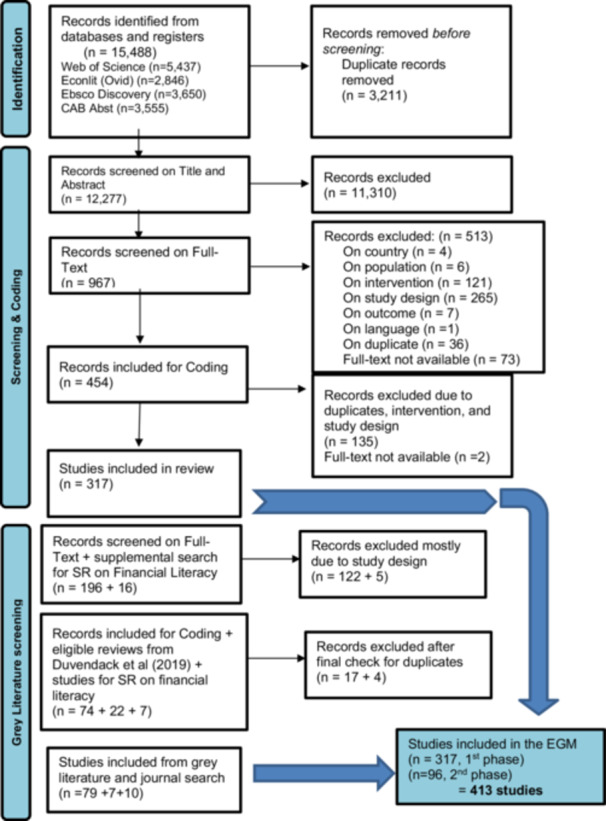
PRISMA diagram.

For the gray literature scanning phase, we screened 196 records on full text, of which 122 were excluded mostly because of study design, resulting in 74 studies for coding and data extraction. Then, we added some systematic reviews from a paper on a review of reviews (Duvendack and Mader 2019), which numbered at 22 studies. After coding or data extraction, 22 were excluded after a final eligibility check for duplicates, leaving a total of 79 studies from gray literature to be included in the map. For the follow‐on systematic review, a supplemental search was executed to gather papers on financial literacy interventions for additional scanning. The team found 16 studies, and after screening and coding stages, 7 studies were included in the map. In addition, we also conducted a journal hand search on the most recent studies for the review and added 10 studies in the map. Combining both phases, a total of 413 studies consisting of impact evaluations (379 studies) and systematic reviews (34 studies) were included in the map. These two phases are presented through a PRISMA diagram (Figure 3).

### 4.2 Overview: Interventions and Outcomes

**Figure 4:**
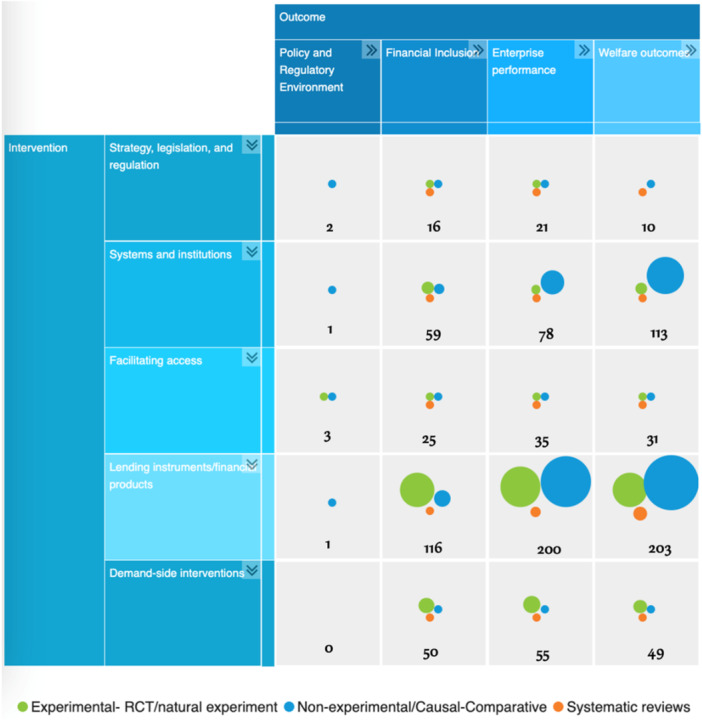
Interactive Evidence and Gap Map by intervention and outcomes, *n* = 413. The numbers in the figure represent the total number of primary studies and systematic reviews per cross‐section. The size of the circle is determined by the number of primary studies and systematic reviews by study design. *Source:* IED and Campbell Collaboration. 2022. MSME Finance Interactive Evidence and Gap Map. Manila and New Delhi.

Figure 4 shows the aggregate map of the five financial sector interventions against the four outcome categories of interest. Most of the evidence included in the EGM is studies examining the impacts of lending instruments or financial products. The most common, and most studied, financial product is microcredit, which we will discuss later. On the other hand, studies that examine policy and regulatory environment as outcomes were scarce. Similarly, there were a few studies examining strategy, legislation, and regulation and facilitating access as interventions. There is a major gap in evidence using demand‐side interventions and examining policy and regulatory environment outcomes, as no study was found on this particular outcome of interest.

The most common intervention is lending instruments/financial products across all firm types. Focusing on the firm types receiving the financial intervention, evidence is largely on microenterprises provided with lending instruments or financial products (274 studies), followed by systems and institutions (132 studies) that support improving access to such financial products and services (Table 4). Moreover, there are relatively more studies about demand‐side interventions targeted at microenterprises compared to other firm types. Community groups have the lowest evidence across all interventions, but we combine community groups with microenterprises. There is no evidence on strategy, legislation, and regulation targeted to community groups.

**Table 4:** Aggregate map, number of studies (primary studies and systematic reviews) by types of firm receiving the intervention and intervention category.

Types of firm receiving the intervention	Strategy, legislation, and regulation	Systems and institutions	Facilitating access	Lending instruments/financial products	Demand‐side interventions
Micro enterprise (incl. single person/household)	17	132	39	274	63
Community group	0	4	2	4	3
Small enterprise	18	15	13	37	12
Medium enterprise	13	9	8	25	4

*Note:* Red = few studies (< 15); Orange = relatively few studies (16–45); Light green = relatively more studies (46–80); Green = many studies (≥ 80); These thresholds are set by the research team for the EGM purpose.

Among the outcomes of interest, welfare outcomes have the greatest amount of evidence, closely followed by enterprise performance and financial inclusion. Welfare outcomes are also targeted mostly to microenterprises among all the firm types. Enterprise performance outcomes are also relatively numerous in terms of microenterprises as well as small enterprises, with 59 studies (Table 5). This also implies that welfare outcomes are typically targeted in microenterprises, while firm performance outcomes are more evident with SMEs. There is a gap in evidence on outcomes relating to policy and regulatory environment across all firm types, with less than 10 studies for all.

**Table 5:** Aggregate map, number of studies (primary studies and systematic reviews) by types of firm receiving the intervention and outcome category.

Types of firm receiving the intervention	Policy and regulatory environment	Financial inclusion	Enterprise performance	Welfare outcomes
Micro enterprise (incl. single person/household)	3	160	247	271
Community group	0	5	5	6
Small enterprise	3	30	59	17
Medium enterprise	2	19	34	6

*Note:* Red = few studies (< 15); Orange = relatively few studies (16–45); Light green = relatively more studies (46–80); Green = many studies (≥ 80); These thresholds are set by the research team for the EGM purpose.

Table 6 presents the distribution of systematic reviews in the EGM that subscribes to the same structure of the general overview of the map as primary studies. Evidence is concentrated on lending instruments or financial products and their effects on welfare outcomes, followed by firm performance outcomes. Second to lending instruments are systems and institutions and demand‐side intervention, with less than 20 systematic reviews. In addition, policy and regulatory environment outcomes and strategy, legislation, and regulation interventions remain scarce in evidence, with only less than five down to no systematic reviews at all.

**Table 6:** Aggregate map on interventions against outcomes filtered by systematic reviews only (total number of systematic reviews = 34).

	Outcomes of interest			
Interventions	Policy and regulatory environment	Financial inclusion	Enterprise performance	Welfare outcomes
Strategy, legislation, and regulation	0	3	3	1
Systems and Institutions	0	13	13	17
Facilitating access	0	5	4	5
Lending instruments/financial products	0	15	19	27
Demand‐side interventions	0	8	7	10

*Note:* Red = few systematic reviews (< 5); Orange = relatively few systematic reviews (6–10); Light green = relatively more systematic reviews (11–15); Green = many systematic reviews (≥ 16); These thresholds are set by the research team for the EGM purpose.

Through this map, we have identified financial literacy interventions as a specific topic for a follow‐on systematic review. This need was identified due to the large number of primary studies on financial literacy present in our initial search and their categorization under demand‐side interventions, which are well‐represented in our EGM (Figure 4, Tables 4 and 6).

All aggregate map tables comprise primary studies and systematic reviews that may be counted in multiple fields in the cross‐section, as some primary studies and systematic reviews have more than one intervention and outcome of interest.

#### 4.2.1 Evidence Base by Intervention (Primary Studies and Systematic Reviews)

The evidence is concentrated on lending instruments/financial products, particularly microcredit (loans), followed by microfinance institutions under systems and institutions, and financial literacy and education programs under demand‐side interventions. Fintech/digital financial services (mobile and e‐money banking and payment systems) were reported to have the most evidence among facilitating access interventions, with around 22 studies. Meanwhile, financial sector legislation and regulations (including tax regime) have the most evidence under strategy, legislation, and regulation. With one paper for each sub‐category: equity, interest‐free bank accounts, and trade credit, several interventions on lending instruments/financial products remain understudied.

In the EGM, there are 30 studies on the strategy, legislation, and regulation interventions, 147 studies on systems and institutions, 45 studies on facilitating access, 347 studies on lending instruments/financial products, and 80 studies on demand‐side interventions (Table 7). These frequencies account for the overlap of firm types in selected papers.

**Table 7:** Primary studies and systematic reviews per interventions category.

Intervention	Number of studies	%
Strategy, legislation, and regulation	30	5%
Systems and institutions	147	23%
Facilitating access	45	7%
Lending instruments/financial products	347	53%
Demand‐side interventions	80	12%

Table 7 shows that there are 347 studies in the map that examined lending instruments/financial products. A total of 163 (47%) of these studies are from Sub‐Saharan Africa, while 129 (37%) are from South Asia. This focus on lending instruments/financial products highlights the microenterprises' need for microcredit that supports their income‐generating activities (Table 8).

**Table 8:** Primary studies and systematic reviews per lending instruments/financial products category.

Lending instrument/financial product	Number of studies
Microcredit (loans)	208
Line of credit	7
Savings	35
Equity (incl crowd financing)	1
Grants and matching grants	51
Supply chain financing	10
Interest‐free bank accounts	1
Trade credit	2
Microinsurance	30
Microleasing/hire purchase	2

The first category of intervention concerns strategy, legislation, and regulation, covering 30 studies. Most of the evidence covers multiple sizes of enterprises; 18 include microenterprises, 19 studies include small enterprises, and 14 studies include medium enterprises. These frequencies account for the overlap of firm types in selected papers. This category covers four specific areas of intervention: (i) national financial inclusion strategy; (ii) financial sector legislation and regulations (including tax regimes); (iii) legal and regulatory framework for payments (including insolvency mechanism); and (iv) financial consumer protection.

**Figure 5:**
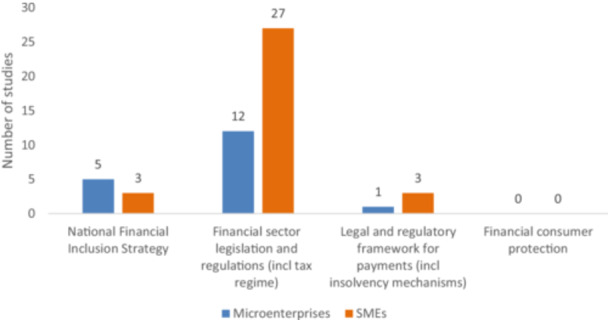
Strategy, legislation, and regulation intervention by firm types (primary studies and systematic reviews).

Figure 5 shows that an overwhelming amount of evidence focused on financial sector legislation and regulations (39 studies), and most of them analyze SMEs (27 studies). There are still relatively few studies related to the national financial inclusion strategy (eight studies) and the legal and regulatory framework for payments (four studies). The EGM did not find any studies examining the impact of financial consumer protection.

**Figure 6:**
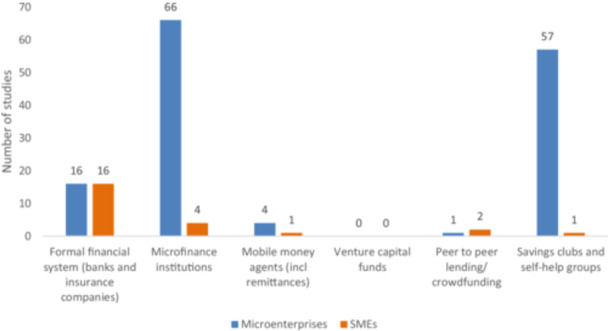
Systems and institutions intervention by firm types (primary studies and systematic reviews).

The second category of interventions is systems and institutions that enable financing. The specific areas of intervention under this category are (i) formal financial system (banks and insurance companies); (ii) microfinance institutions; (iii) mobile money agents (including remittances); (iv) venture capital funds; (v) peer‐to‐peer lending/crowdfunding; and (vi) savings clubs and SHGs. The EGM found a total of 147 studies under this category. As Figure 6 shows, most of the evidence under this category examines the impact of microfinance institutions (70 studies), most of which are targeting microenterprises and looking at welfare outcomes. This indicates the growing popularity of microfinance institutions—and microcredit, which will be discussed in the next section—over the past two decades.

Next, the EGM found a significant number of evidence on savings clubs or SHGs (57 studies). SHGs are a community‐based intervention that has been found to have positive effects.^4^ Some of these positive outcomes for women are on savings and assets, political empowerment, and health (e.g., contraceptive use), but not on self‐empowerment. However, the ways in which these empowerment benefits are achieved are possibly through group work and capacity development, rather than necessarily savings and business activities. While women use such clubs deliberately to keep liquid resources out of the hands of their partners, the evidence does not suggest that SHG participation increases intra‐household conflict.

There is much evidence on formal financial systems (banks and insurance) targeting both microenterprises and SMEs. While studies on microfinance and savings clubs are mainly examining welfare outcomes, studies on formal financial institutions mostly look at the impact of enterprise performance. The EGM also found a small number of studies in two areas that are growing: mobile money (five studies) and peer‐to‐peer lending (three studies). However, no studies on venture capital are eligible for the EGM (Figure 6).

The third category of interventions concerns facilitating access to finance. There are 45 studies included in this category. Microenterprises are covered in 41 studies; small enterprises were covered in 14 studies, and medium enterprises were covered in 9 studies. The specific areas of interventions under this category are: (i) bank linkages; (ii) pitching events^5^; (iii) competitions^6^; (iv) credit guarantees; (v) digital financial services/financial technology; (vi) informal financial agencies or agents; (vii) collateral registries; and (viii) credit reporting systems.

**Figure 7:**
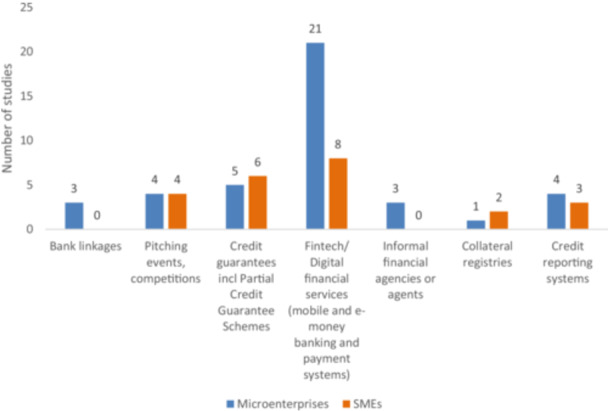
Facilitating access intervention by firm types (primary studies and systematic reviews).

As Figure 7 shows, most of the included studies are concerned with the impact of digital financial services in facilitating access to finance to both microenterprises and SMEs.^7^ Consistent with the discussion in the earlier paragraph, evidence analyzing SMEs (eight studies) focused on enterprise performances, while studies analyzing microenterprises are mostly examining welfare outcomes. There is also a significant number of studies (11 studies) on credit guarantee schemes; most of which focus on the performance of SMEs. The EGM also found seven studies on credit reporting systems; four of which analyzed the impact on microenterprises, and three on SMEs. Evidence on collateral registries (three studies) examined institutional capacity and firms being established or survival of firms as outcomes for SMEs. Meanwhile, there were three studies on bank linkages and three studies on informal financial agents, which focus on the input adoption practices, financial inclusion, and economic outcomes of microenterprises.

Provision of lending instruments and financial products is the fourth category of intervention that the EGM considers. Lending instruments and financial products is the area where most of the evidence is available, with 347 studies on the topic. Ten areas of intervention under this category are included: (i) microcredit (loans); (ii) line of credit; (iii) savings; (iv) equity (including crowd financing); (v) grants and matching grants; (vi) supply chain financing; (vii) interest‐free bank accounts; (viii) trade credit; (ix) microinsurance; and (x) micro leasing/hire purchase.

**Figure 8:**
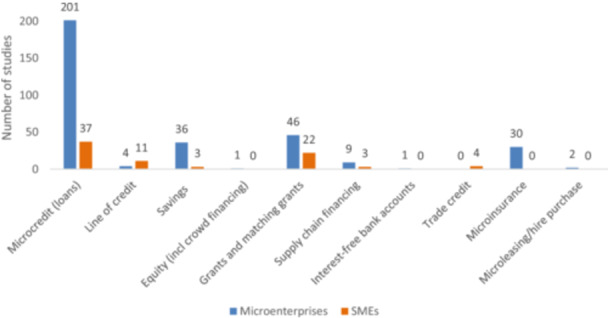
Lending instruments/financial products intervention by firm types (primary studies and systematic reviews).

As shown in Figure 8, the most common financial instrument/product is microcredit (238 studies). Most of the evidence looks at the impact of microcredit on microenterprise (201 studies), focusing on welfare outcomes (economic aspect, gender, and education). Studies examining the impact of microcredit on SMEs (37 studies), on the other hand, focus more on firm performance (employment and wages, sales, revenue, and profit, and management practices).

Grants (68 studies) and savings (39 studies) are among the financial instruments that have been extensively researched, whereas other financial products have not received as much attention, namely: credit line (15 studies), microinsurance (30 studies), supply chain financing (12 studies), and trade credit/financing (4 studies). SME studies on the impact of credit lines commonly examine outcomes on employment and wages, sales, revenue, profit, and management practices. Microinsurance studies are highly concentrated on economic outcomes, input adoption practices, and access to financial services. Evidence on supply chain financing of microenterprises looks mostly at sales, revenue, and profit. Two of the studies were on microleasing, the other intervention that was considered in the map.

The fifth and final category is demand‐side interventions. This includes (i) financial literacy and education programs; (ii) digital literacy; and (iii) opening bank accounts for entrepreneurs. The EGM includes 80 studies under this category.

**Figure 9:**
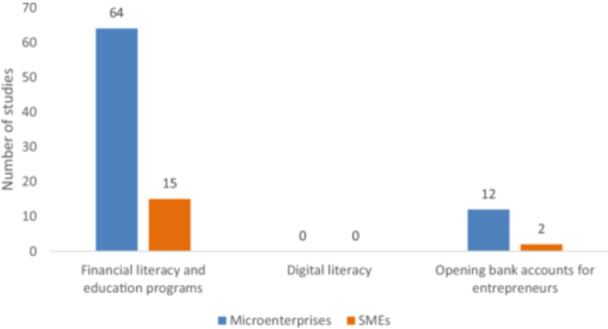
Demand‐side intervention by firm types (primary studies and systematic reviews).

Figure 9 shows that the majority of evidence on the demand side is studies on financial literacy and education programs (79 studies), mostly targeting microenterprises. A review of financial literacy interventions—which are mostly conducted for personal finance rather than for MSMEs—found positive effects on both financial knowledge and behavior, such as budgeting. There was also increased use of credit savings, though the effect was small. Effects on financial knowledge appear to be sustained, though changes in financial behavior are less so.

The EGM found 14 studies looking at the impact of opening bank accounts for entrepreneurs, almost all of which analyze microenterprises. The outcomes of interest are concentrated on active use of credit/banking services under financial inclusion outcomes and still sales/revenue, as well as profit and exports under firm performance. Despite the growing use of digital financial services and a lot of reported problems regarding their use (such as irresponsible borrowing or investments), no studies on promoting digital literacy were found to be eligible to be included in the map.

#### 4.2.2 Evidence Base by Outcome Category and Sub‐Category

**Figure 10:**
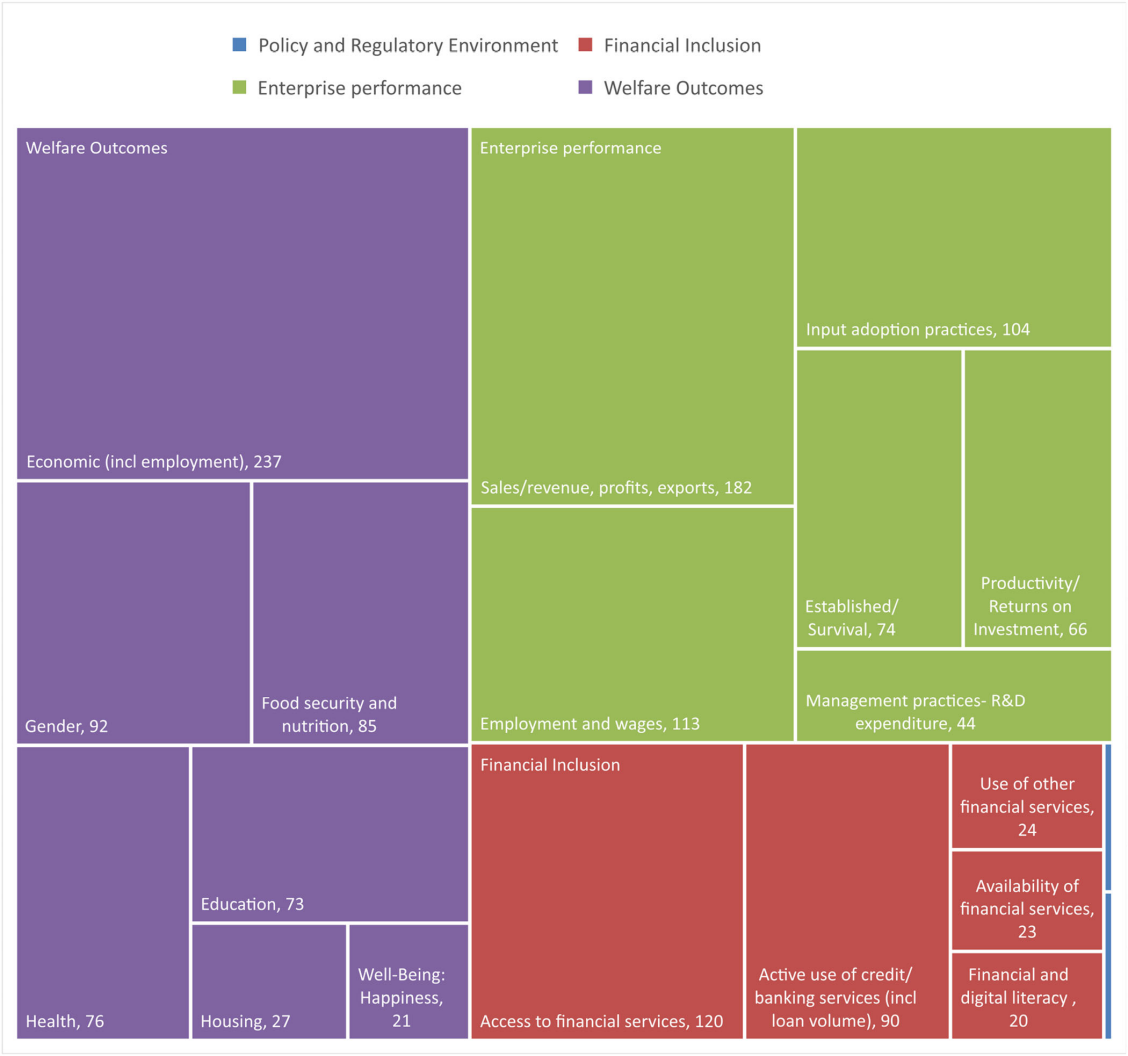
Summary of outcome categories and sub‐categories.

In terms of outcome categories and sub‐categories, the evidence is concentrated on welfare outcomes, particularly economic outcomes. This is followed by two sub‐categories under firm performance, namely: sales or revenue, profits, and exports, and employment and wages outcomes (Figure 10). Among financial inclusion outcomes, access to financial services was reported to have the most evidence with greater than 100 studies, more than the evidence on employment and wages under firm performance. Outcomes on policy and regulatory environment remain to be understudied, with two papers (two thin blue boxes in Figure 10) each for sub‐categories: regulatory framework and institutional capacity.

#### 4.2.3 Secondary Dimensions of the Map

##### 4.2.3.1 Study Design

**Figure 11:**
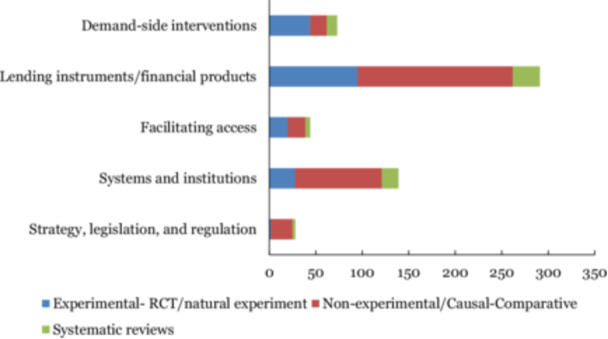
Number of primary studies and systematic reviews as per the study design and intervention sub‐categories.

Figure 11 presents the distribution of included studies by study design and intervention group. Of the 413 studies included in the map, 243 primary studies employ non‐experimental or quasi‐experimental designs—mostly propensity score matching and instrumental variable methods—while 136 primary studies used experimental methods, and 34 were systematic reviews.

Approximately 8.2% (34 out of 413) of the included studies are systematic reviews, which is comparatively lower than other EGMs.

##### 4.2.3.2 Region and Country

By region, 175 studies (43%) provided evidence from Sub‐Saharan Africa, 142 studies (35%) from South Asia, 86 studies (21%) from East Asia and the Pacific, 66 studies (16%) from Latin America and Caribbean, 28 studies (7%) from Europe and Central Asia, and 21 studies (5%) from Middle East and North Africa. Most of the included evidence covers lower‐middle income countries (66%) and low‐income (26%), and to a lesser extent, upper‐middle‐income countries (26%).

**Figure 12:**
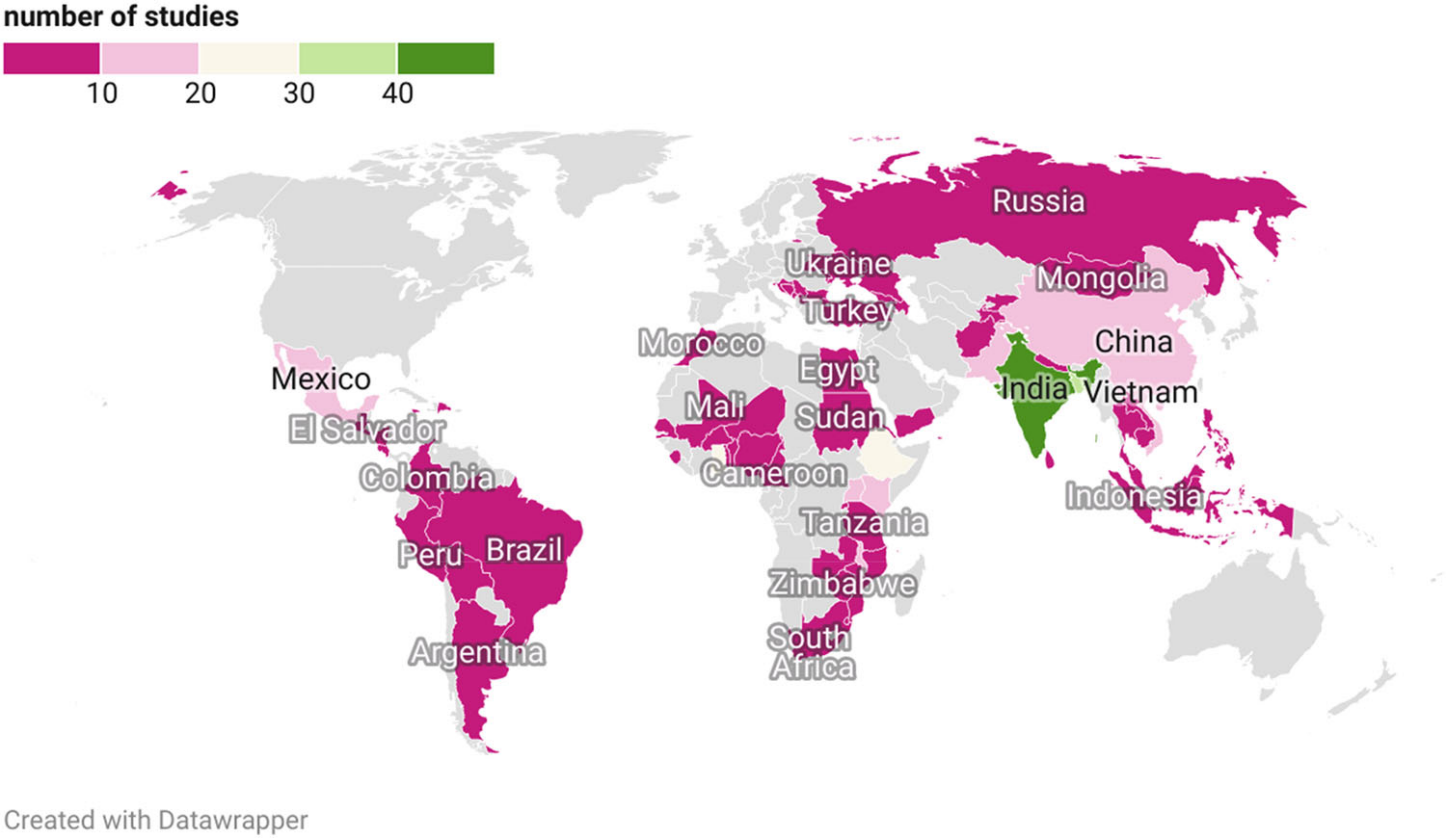
Geographical map of the number of primary studies and systematic reviews by country.

A geographical map illustrating the distribution of both primary studies and systematic reviews is presented below (Figure 12). For systematic reviews, the country was determined based on the locations mentioned in the articles discussed within the review. This ensures consistency and a clear representation of the geographical origins of the articles included in our analysis.

India is the most common country or setting among studies included in the map, with 50 studies, followed by Bangladesh (36 studies), Ghana (29 studies), Ethiopia (23 studies), and Kenya with 19 studies (Figure 12). Evidence in South Asia and the Sub‐Saharan Africa region is usually about savings groups, microcredit, and microfinance institutions. There are also numerous studies conducted in countries like China (18 studies), Vietnam (15 studies), Pakistan and Uganda (both 13 studies), as well as Mexico and Malawi (both 12 studies).

**Figure 13:**
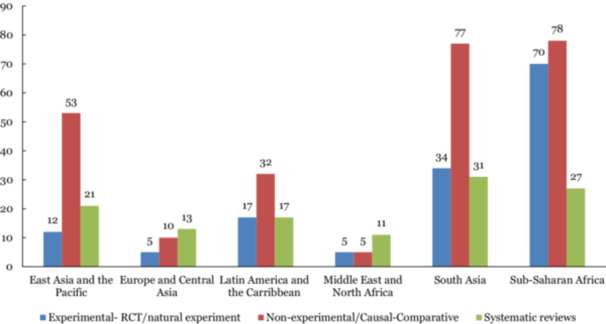
Number of primary studies and systematic reviews by study design and by region.

*Region‐wise study design:* In most regions, non‐experimental primary studies were dominant, especially in Sub‐Saharan Africa and South Asia. For Sub‐Saharan Africa, which had the greatest number of included studies in this EGM, the number of experimental primary studies was comparatively high. On the contrary, experimental primary studies tend to be scarcer in other regions, such as East Asia and the Pacific and Europe and Central Asia (Figure 13).

##### 4.2.3.3 Authors

Table 9 below shows the list of the most common authors included in the map. We have sorted the studies alphabetically and have identified 45 authors with 2 or more studies. Then we reviewed all 45 to check other studies in which they are co‐authors (meaning not first authors) and generated the table of authors with 5 studies or more included in the map.

**Table 9:** Most common authors in the EGM.

Authors	Number of included studies in the map
Karlan D.	23
McKenzie D.	21
De Mel S.	9
Lensink R.	8
Gine X.	8
Banerjee A. V.	8
Dupas P.	7
Imai K. S.	7
Augsburg B.	6
Khandker S. R.	6
Blattman C.	5
Bruhn M.	5
Crépon B.	5
Field E.	5

##### 4.2.3.4 Population Groups

**Figure 14:**
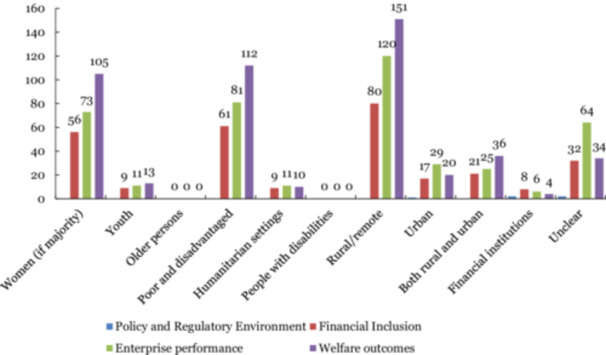
Distribution of population group by outcome.

The effects of financial inclusion interventions on MSMEs tend to cover welfare outcomes the most across almost all population groups. This is followed by enterprise performance and financial inclusion outcomes. A notable exception is studies that cover the urban population, considering that most of these studies look at enterprise performance rather than welfare outcomes (Figure 14).

## 5 Discussion and Gaps in Evidence

### 5.1 Summary of Main Results

The map is based on 413 studies, consisting of 379 primary studies and 34 systematic reviews. Most of the studies (379 studies) targeted microenterprises, particularly households and smallholder farmers; 7 studies target community groups, while 109 studies target SMEs. A total of 147 studies targeted multiple firm sizes.

Lending instruments and financial products are the type of intervention that is most studied, while very few studies examine interventions pertaining to strategy, legislation, and regulation for improving access to finance.

An overwhelming number of studies are on microcredit/loans (238 studies). This reflects the growing popularity of this area of study. However, the evidence suggests that microcredit has not been proven to be the pathway out of poverty, as hoped. This is based on the aggregated evidence from primary studies and reviews, which collectively suggest that the impact of microcredit on poverty alleviation has been limited, especially for women, the poor, and the most disadvantaged populations, who have benefited less than expected. Financial literacy interventions—which are mostly conducted for personal finance rather than for MSMEs—have substantial evidence as having an effect on both financial knowledge and behavior, such as budgeting. Emerging financial interventions, such as facilitating access to financial technology/digital financial services, are relatively understudied. Despite their potential to increase financial inclusion by providing innovative solutions to unbanked populations, especially in remote and underserved areas, these interventions have not been prominently explored in the existing literature. Similarly, some financial instruments, such as credit lines, supply chain financing, and trade financing, also have very few studies.

Evidence on welfare outcomes is predominant in the literature, followed by enterprise outcomes. There is a major evidence gap in policy and regulatory environment outcomes.

Moreover, we have observed a difference in the outcomes of interest between evaluations focusing on SME and microenterprises. SME evaluations tend to investigate firm performance, with less focus on the welfare effects on owners and employees, including poverty reduction. In contrast, the micro‐enterprise literature has investigated welfare outcomes, with less focus on aspects of firm performance. Some studies on microenterprises tend to look on cash flows and profitability, but issues with management and low returns arise.

By region, Sub‐Saharan Africa takes up the largest share of evidence with 175 studies, followed by 142 studies from South Asia. Several studies also investigate rural populations or populations in remote areas, with 192 studies, 126 studies on poor and disadvantaged, and 114 papers on women.

### 5.2 Summary of Findings From Selected Reviews in the Map

This thematic synthesis of reviews was developed through the collection of reviews, reading and extraction, focus and scope, and summary contents. This synthesis came from the selected systematic reviews included in this map. The focus and scope are on evaluating financial inclusion interventions aimed at supporting productive activities by MSMEs. This included microcredits, grants, microinsurance, savings products, and entrepreneurial training, such as business and financial literacy training and SHGs. Here, we considered emerging themes like new information, the most effective finance instrument, impacts on gender, and evidence‐based. Reviews with methodological limitations were identified, and findings were presented cautiously as the risk of bias was not conducted. The summary highlighted the most common and extensively studied financial interventions, such as microcredit. The summary aimed to provide a comprehensive and nuanced understanding of the existing systematic reviews on financial inclusion interventions for MSMEs, while transparently addressing the methodological considerations as published in the protocol.

The lack of access to finance is one of the most significant hurdles to MSME development in LMICs. There are a variety of interventions targeted at MSMEs to solve the issues in accessing financial services and products. These interventions include supporting financial intermediaries, directly providing financial products and services or otherwise facilitating access to them, and demand‐side interventions. The most common and most studied financial intervention is microcredit. Microcredit is often supplied for purposes other than enterprise development. This summary is restricted to studies of financial inclusion interventions intended to support productive activities by MSMEs.

The 16 systematic reviews summarized here include evaluations covering life skills and financial products such as (micro)credits, grants, microinsurance, and saving products targeting (M)SMEs, as well as entrepreneurial training (i.e., business and financial literacy training and SHGs as a community approach).

#### 5.2.1 What We Have Learned

##### 5.2.1.1 Improving Access to Finance Interventions

Microcredit to households has not been proven to be the pathway out of poverty as hoped. Overall, studies find that while suitably priced microfinance often leads to micro‐enterprise creation or expansion, there is not a corresponding increase in household income and consumption. One paper combining findings from seven studies concludes that “the impact on household business and consumption variables is unlikely to be transformative and may be negligible,” (Cui et al. 2013). Moreover, both credit and savings schemes for poor individuals and households are as likely to use support and smooth consumption—including catastrophic expenditures, notably health‐related—or purchase of household items as they are for production.

In contrast, providing access to finance for SMEs positively affects some aspects of firm performance. A systematic review covering 15 studies of interventions that provided only SME finance, including several credit types, guarantees, grants, priority‐lending regulations, and overdraft facilities (Kersten et al. 2017), shows that improving access to finance improves capital investments, revenue, and employment creation. However, there is no effect on productivity or wages. Generally, the impact increases with firm size. But larger firms are less likely to be credit‐constrained, so the challenge is to lend to businesses large enough to benefit, but not so large as to have alternative sources of finance.

##### 5.2.1.2 Which Finance Instrument Is (Most) Effective?

Providing finance in combination with other business support services has a larger impact than finance alone, though the evidence is mostly about micro‐enterprises rather than SMEs.

A meta‐analysis of 23 quantitative studies on entrepreneurship programs (Cho and Honorati 2014) found evidence that when combined with a training program, (in‐kind) grants have a greater impact on poor households and their businesses than microcredit approaches. In line with that, summary evidence on seven RCTs, including microcredit interventions, failed to prove significant results in terms of the expected transformative effect on poor households. It shows that microcredit has a larger positive effect on households with previous business experiences, but also that already moderate heterogeneity results in different impacts. Indeed, it should be considered that microcredit may potentially cause negative effects, as one RCT provided evidence of negative potential side effects (Meager 2019), like over‐indebtedness and consequences for livelihoods. However, this issue was addressed by too few evaluations to conclude these potential negative side effects.

After the initial enthusiasm for microcredit, the view has emerged that support for savings, especially through savings groups, may be the more appropriate way to reach and benefit poorer households. A systematic review and meta‐analysis covering 24 studies on saving promotion interventions in Sub‐Saharan Africa (Steinert et al. 2018) shows that the interventions can significantly increase saving amounts and enterprise investment decisions, while also having positive effects on poverty reduction measures like household consumption returns from family businesses, though small in size. The review provides evidence that supply‐side saving interventions (e.g., providing access to formal saving accounts, mobile money saving schemes, and others) are more effective than demand‐side interventions, including educational or motivational elements to promote savings.

A review of financial literacy interventions—which are mostly conducted for personal finance rather than for MSMEs—found positive effects on both financial knowledge and behavior, such as budgeting. There was also increased use of credit savings, though the effect was small. Effects on financial knowledge appear to be sustained, though changes in financial behavior are less so.

While entrepreneurship training alone increases knowledge, it does not generally lead to new businesses being set up. Matching grants are among the forms of business support that are found to improve firm performance—in contrast to export promotion and innovation programs, which were not proven to do so. The effects of matching grants, however, are small.

#### 5.2.2 Impacts on Gender‐Related Topics

Financial inclusion has generally delivered small effects at best, which are mostly on lower‐level outcomes rather than sustained improvements in women's income or empowerment. A review of 25 studies on the effects of microcredit on women's control over household resources (Vaessen et al. 2014) found, at best, small and statistically insignificant effects.

Many interventions to support female‐owned MSMEs have failed because of social norms of women's roles in society and the corresponding household gender roles. Providing financial resources such as business grants and microcredit to women has often been found to have no effect on enterprise profitability for their enterprises, whereas there are positive effects for men. However, it has been argued that this is because the resources directed to women are used for men's enterprises. Studies do find positive effects on income for women in female‐headed households, or where the woman has a good relationship with their spouse.

Overall, providing financial resources alone without paying attention to gender‐related constraints will often not improve business performance or women's agency. Mechanisms that may increase women's direct control over resources include providing resources in private, paying directly to a woman's bank account, or using digital payment systems.

Generally, multicomponent programs, such as the BRAC graduation model, have proved to be more promising both in terms of sustained improvements in income and empowerment, most notably self‐empowerment, which is also called the power within. The graduation model combines an asset or cash transfer with financial literacy and vocational training and other access to financial services, with health and education interventions and activities to directly enhance social empowerment. Even business training, while not consistently improving business outcomes, has been found to have positive effects on personal and social empowerment.

Self‐help groups or SHGs are a community‐based intervention that has also been found to have positive effects. A review of 23 impact evaluations (Brody et al. 2015) reported positive effects on economic and political empowerment as well as mobility and contraceptive use. SHGs were also found to have positive effects on economic measures such as savings and assets, but not on self‐empowerment. However, the ways in which these empowerment benefits are achieved are possibly through group work and capacity development, rather than necessarily savings and business activities. While women use such clubs deliberately to keep liquid resources out of the hands of their partners, the evidence does not suggest that SHG participation increases intra‐household conflict.

Originating in India and increasingly used in Sub‐Saharan Africa, SHGs have long functioned as savings clubs but also provide a broader range of functions, such as “transport committees” that help women in labor get to a delivery facility. A review of 47 studies of SHGs across Africa and Asia (Gugerty et al. 2019) found that health‐related areas had the most consistently positive impact. But, where it was considered, the record of groups involving the very poor was mixed.

Finally, a specific area of success is providing business support to sex workers. By providing them with an alternative source of income, such interventions have been found to reduce unsafe sex. However, effects are less clear for non‐sex workers, and no positive effect was found in reducing intimate partner violence (though it does not increase it, as is sometimes feared).

#### 5.2.3 A Comment on the Evidence Base

The literature can be divided into studies of SME finance and support for micro‐enterprises, which are mainly household or single‐person enterprises, including agriculture and non‐farm rural enterprises. SME evaluations have relied less on RCTs than have studies of micro‐enterprises. Moreover, they have focused on firm performance, with little attention to employment and the welfare effects on owners and employees, including poverty reduction. In contrast, the micro‐enterprise literature has focused on welfare outcomes, while neglecting some aspects of firm performance. It would be beneficial to have studies of MSMEs that collect indicators across the causal chain.

Interpreting study findings is complicated by the fact that financial interventions are often bundled with other services, such as training. Factorial designs, which offer different bundles and so allow an analysis of their relative effectiveness, are comparatively rare and would be a very welcome and useful addition to this literature.

### 5.3 Areas of Major Gaps in the Evidence

As previously mentioned, two major gap areas are (1) strategy, legislation, and regulation as intervention, and (2) policy and regulatory environment outcomes. As popular interventions are usually lending instruments like microcredit or loans and systems and institutions that provide microfinance, there is relatively little evidence on demand‐side interventions, especially targeted to SMEs. In line with this, some gaps in outcomes are relating to financial and digital literacy and the use of other financial services. Well‐being and housing outcomes are also relatively understudied.

With regard to region, potential study settings that should be explored further are the Middle East and North Africa, Europe and Central Asia, and Latin America and the Caribbean. There is also a major gap in studies about people with disabilities and their access to financial interventions or involvement with MSMEs per se. Studies targeting youth, urban population, and humanitarian settings are also relatively underrepresented.

There is limited systematic review evidence on interventions facilitating access, and limited experimental evidence on systems and institutions as interventions.

The gaps in the map depend on how different types of firms are impacted by the intervention, which narrows its scope. There is a need to further explore the impacts of interventions related to financial literacy, digital services and other financial products tailored to SMEs.

### 5.4 Potential Biases in the Mapping Process

We worked with an information specialist who specializes in information access and retrieval to develop our search strategy. The search terms we utilized, on the other hand, were created in consultation with our advisory team, which is composed of a number of MSME finance experts. The search strategy was comprehensive with no time restriction on the published literature, but only English language results were included. Duplicate screening was implemented, with a third‐party arbitrator to reconcile disagreements.

Given the extensive literature on the topic, it is however possible that our search strategy missed some studies on MSME financing interventions, especially those that were published in non‐English languages. To eliminate potential bias, we also cross‐referenced the lists of papers that were included in eligible systematic reviews.

### 5.5 Limitations of the EGM

Our EGM may over‐represent MSME financing interventions studies that used non‐experimental or quasi‐experimental designs, as majority of eligible studies used propensity score matching or instrumental variable techniques. By doing citation tracking for the systematic reviews of RCTs, we reduced the possibility of over‐representing non‐experimental studies. Studies were likewise restricted to those published in English. In addition, publications and reports with multiple interventions or multiple outcomes may have appeared in multiple quadrants of the map. This is crucial to keep in mind when analyzing the map. The research team also scanned the gray literature, but given the vast literature on financial interventions, some studies may have been overlooked.

While this EGM provides a comprehensive overview of studies on MSME financing, it does not include a risk of bias assessment, as the primary purpose of this EGM is to map the existing evidence base. Meanwhile, a thematic synthesis was performed to provide a general picture of the emerging themes found in the included systematic reviews.

### 5.6 Stakeholder Engagement Throughout the EGM Process

We formed an advisory group made up of professors and researchers from various finance‐related organizations and MSME finance interventions. Representatives from the Alliance for Financial Inclusion, a policy organization, and UNESCAP, a development organization, are among them. We also spoke with authors of financial inclusion and microcredit research projects to gain their valuable insights. In early 2022, we had an online exploratory meeting with experts in the field to solicit feedback on the development of our EGM framework. The EGM is prepared in close collaboration with the IED of the ADB. We have solicited some comments on our EGM through a webinar held on July 26, 2022.

## 6 Authors' Conclusions

The map has addressed the authors' objectives, which are to:

- Generate an online interactive map and other versions that showcase the existing evidence on the impacts of interventions on MSMEs' access to finance;
- Describe key messages and summaries of evidence as well as gaps across study designs, contexts, geographical location, firm sectors, and population sub‐groups; and
- Identify focus area/s for a follow‐on systematic review.

### 6.1 Implications for Research, Practice, and/or Policy

The EGM confirms known evidence and presents major gaps in both impact evaluations and systematic reviews. The following are some policy and research implications:

- There is a need to further explore strategy, legislation, and regulatory interventions, which may affect financial intervention.
- Potential research focus can be toward interventions targeting SMEs and looking at policy and regulatory environment outcomes, and welfare outcomes sub‐categories like food security and nutrition, health, and gender outcomes. One of the major gaps is on demand‐side interventions and their impacts on policy and regulatory environment, as well as facilitating access interventions and impacts across all outcomes (since studies for this are relatively few and fairly distributed study designs). Interventions facilitating access seem to be emerging because of the connection with technology, thus an interesting topic to explore further. Specifically, given the increasing attention and rapid development of digital financial services, future research should prioritize these types of interventions. To date, most of the research is conducted in Sub‐Saharan Africa and South Asia. Further research in other regions could be conducted to allow a more holistic understanding of the effects of financial inclusion interventions.
- The map should be updated annually as evidence continuously piles up on the various financial sector interventions and even innovative programs that improve MSMEs' access to finance.

## Author Contributions

The lead author is the person who developed and co‐ordinated the EGM team, discussed and assigned roles for individual members of the team, liaised with the editorial base and took responsibility for the updates of the EGM. Content expertise: Prof. Robert Lensink is a professor of finance and financial markets at the University of Groningen. Alyssa Villanueva is a consultant at the Asian Development Bank and has worked on projects related to SME credit guarantee schemes and SME lending schemes. Systematic review method expertise: Most of the authors are experienced systematic reviewers, which means that they are proficient in conducting various processes in an EGM, such as screening, quality assessment and coding. Howard White provided technical support for conducting the review. EGM methods expertise: Howard White provided technical and strategic support for the development of EGM in the Campbell library. Most of the team members have previous experience in systematic review methodology, including search, data collection, statistical analysis, and theory‐based synthesis, which means that they are proficient in carrying out the various processes in an EGM, such as search, eligibility screening, quality assessment and coding. Information retrieval expertise: The authors are supported by an information retrieval specialist, Dr. John Eyers, on an as‐needed basis. John Eyers is a trained information retrieval specialist and has experience in supporting over 50 systematic maps and reviews in social science areas.

## Conflicts of Interest

The authors declare no conflicts of interest.

## Plans for Updating the EGM

We plan to update the map (or support others in doing so) when sufficient further studies and resources become available.

## Differences Between Protocol and Map

None.

## Sources of Support

Asian Development Bank provides financial support to this EGM under a technical assistance TA‐6701 REG: Supporting Evaluations for Development Effectiveness in Asia and the Pacific, 2021–2022 (Subproject 1): Evidence Gap Map and Systematic Review on the Impact of Access to Finance Interventions on MSMEs (project number 53354‐002).

## Peer Review

The peer review history for this article is available at: https://www.webofscience.com/api/gateway/wos/peer-review/10.1002/cl2.70061.

## Supporting information

MSME_Finance_EGM_online_supplements_or_appendix.

## Endnotes

1 The country by income distribution follows the World Bank Income Classification. The income groupings are based on World Bank's income classification of countries. GNI per capita of the income groups included in this study are as follows: (1) low‐income ($1045 or less), (2) lower‐middle income ($1046–$4095), and upper‐middle‐income ($12,696 or more).

2
https://onlinelibrary.wiley.com/doi/full/10.1002/cl2.1341.

3 Independent Evaluation Department (2021). Evaluation Approach Paper: Evidence and Gap Map and Systematic Review on the Impact of Access to Finance Interventions on Small and Medium‐Sized Enterprises. Manila: Asian Development Bank.

4 SHGs originated in India, but the model is being increasingly used in sub‐Saharan Africa. Savings clubs are already well‐established, as are women's groups with a broader range of functions, such as “transport committees” which help women in labor get to a delivery facility.

5 Pitching events help entrepreneurs evaluate their businesses, the commercialization concept, and expected revenue streams, as well as undertake a market sizing exercise and characterize their competitive positioning.

6 Competitions are contests where entrepreneurs pitch their business plans to win funding for their start‐ups.

7 Many studies on mobile payments tend to be excluded due to population groups such as targeting consumers not related to MSMEs or the intervention is not for productive or business purposes.
